# Escherichia coli: A Rare Cause of Prosthetic Valve Endocarditis

**DOI:** 10.7759/cureus.38402

**Published:** 2023-05-01

**Authors:** Diego P Peralta, Aymara Y Chang

**Affiliations:** 1 Division of Infectious Diseases, Texas Tech University Health Sciences Center, El Paso, USA; 2 Internal Medicine, Texas Tech University Health Sciences Center, El Paso, USA

**Keywords:** prosthetic valve endocarditis, mitral valve, prosthetic valve, infective endocarditis, escherichia coli

## Abstract

Prosthetic valve endocarditis (PVE) is a complication of valve replacement associated with high morbidity and mortality. *Escherichia coli *(*E. coli*) is rarely associated with infective endocarditis (IE), accounting for less than 1% of the cases reported. The low frequency is attributed to the organism’s low affinity to adhere to the endocardial endothelium. Risk factors that may play a role in developing IE by *E. coli* include age above 70, diabetes mellitus, a genitourinary source of infection, female sex, healthcare contact, implanted endovascular devices, and immunosuppression. We present a 70-year-old Hispanic woman who arrived with constitutional symptoms and persistent urinary complaints. She had diabetes mellitus, recurrent urinary tract infections, and native mitral valve IE treated with antibiotic therapy and tissue valve replacement. The valve was replaced with a mechanical valve a second time due to malfunctioning. The patient was found to have *E. coli *bacteremia and ultimately diagnosed with PVE. She was treated solely with ceftriaxone and gentamicin combination therapy resulting in complete resolution of valve vegetations. Our case represents the 11th report of this uncommon disease and illustrates its epidemiology and associated risk factors. We summarize the previous 10 cases reported and highlight the lack of prospective trial data to define optimal therapy for managing PVE caused by *E. coli*.

## Introduction

Infective endocarditis (IE) is an infection of the endocardium and heart valves. The characteristic finding of IE, a vegetation, comprises a collection of platelets, fibrin, microorganisms, and inflammatory cells attached to the endocardium [1]. It commonly involves heart valves but may also occur at a septal defect site, on a chordae tendineae, or the mural endocardium [1]. The most common pathogens related to IE include staphylococci, streptococci, enterococci, and the “HACEK” organisms (*Haemophilus* spp., *Aggregatibacter* spp., *Cardiobacterium hominis*, *Eikenella corrodens*, and *Kingela* spp.) [1,2]. In contrast,* Escherichia coli* (*E. coli*) has been identified as a rare cause of IE, accounting for only 0.51% of the cases [3]. It has been attributed to the organism’s lack of traditional virulence factors resulting in its inability to adhere to the endocardium [4]. Reported risk factors for *E. coli* IE include a genitourinary infection, diabetes mellitus, female sex, previous healthcare contact, implanted endovascular devices, immunosuppression, and age above 70 [5,6]. Treatment consensus for managing IE caused by *E. coli *is lacking; however, combination antimicrobial therapy for six weeks is reasonable unless the disease is refractory to antibiotics. Then, surgical intervention is recommended [2].

## Case presentation

A 70-year-old Hispanic woman presented with a one-week history of subjective fever, chills, night sweats, suprapubic pain, persistent dysuria, and new onset confusion. There were also reports of progressive generalized weakness, fatigue, and decreased appetite for several weeks. Two weeks before, the patient was discharged from the hospital after being treated for a urinary tract infection caused by *E. coli*. The patient revisited the emergency room ten days after due to persistent dysuria and urinary frequency despite the completion of recommended antibiotic therapy. With no other concerns noted on labs and imaging, the patient was discharged home to complete a five-day course of oral cefuroxime after receiving one dose of intravenous ceftriaxone while in the emergency room.

The patient had type 2 diabetes mellitus, hypertension, dyslipidemia, chronic kidney disease stage III, congestive heart failure, and recurrent urinary tract infections. She was treated for culture-negative native mitral valve IE with empiric vancomycin, ceftriaxone, gentamicin, and bioprosthetic valve replacement, #29 St. Jude Biocor® tissue valve, eight years before. The bioprosthetic valve was replaced with a mechanical valve, #27 St. Jude mechanical mitral prosthesis, five years after the initial intervention due to the development of severe valvular stenosis.

On admission, the evaluation revealed an obese woman (BMI 31.3) with a temperature of 39.4^o^C, a heart rate of 78 bpm, a respiratory rate of 17 bpm, and a blood pressure of 140/68 mmHg. She remained drowsy throughout the examination but was arousable to stimuli and commands. During cardiac auscultation, a systolic murmur and a click were heard loudest in the mitral area. The abdominal exam was positive for pain in the suprapubic area, but no peritoneal signs. There was no tenderness in the costovertebral angles, and no endocarditis stigmata were evident on examination.

Initial laboratory tests were significant for hyperglycemia, thrombocytopenia, transaminitis, and elevated BUN and creatinine. The urinalysis was abnormal; however, the urine sample had poor quality. The cerebrospinal fluid analysis was unremarkable (Table 1).

**Table 1:** Initial blood and urine workup results hpf: high power field, lpf: low power field

	Normal Range	Results
Blood Tests		
White blood cells	4.5-11.0 x 10^3^/μL	7.55 x 10^3^/μL
Red blood cells	3.5-5.5 x 10^6^/μL	4.66 x 10^6^/μL
Hemoglobin	12.0-15.0 g/dL	12.6 g/dL
Platelets	150-450 x 10^3^/μL	115 x 10^3^/μL
Sodium	135-145 mmol/L	134 mmol/L
Potassium	3.5-5.1 mmol/L	4.2 mmol/L
Chloride	98-107 mmol/L	101 mmol/L
Bicarbonate	22-30 mmol/L	23 mmol/L
Glucose	74-106 mg/dL	209 mg/dL
Blood urea nitrogen	7-17 mg/dL	24 mg/dL
Creatinine	0.52-1.04 mg/dL	1.2 mg/dL
Glycated hemoglobin A1c	< 5.7 %	8.5 %
Urine Tests		
Nitrate	Negative	Negative
Leukocyte esterase	Negative	Small
White blood cells	0-3/hpf	3-5
Red blood cells	0-3/hpf	0-1
Bacteria	Negative	Negative
Squamous epithelial cells	0-1+/lpf	2+

Chest radiograph showed the prosthetic mitral valve but no evidence of acute cardiopulmonary disease. Previous admission CT scan abdomen/pelvis with contrast showed multiple bilateral renal cysts and thickening of the endometrium. There was no evidence of hydronephrosis, hydroureter, or renal stones. However, an abdominal ultrasound reported a non-obstructing 0.3 cm renal calculus within the right kidney.

The patient was started on empiric vancomycin and meropenem after collecting two blood culture sets to treat possible sepsis of genitourinary origin. Blood culture sets were processed using the BD BACTEC™ FX blood culture system. On day two, blood cultures became positive for a gram-negative rod (GNR). Per protocol, a blood sample was plated in Chocolate, CDC, MacConkey, and Blood agars. All agars started to show bacterial growth within the next 24 hours. Lactose-fermenting GNR colonies were seen in the MacConkey agar plate, later identified as* E. coli *by the BD™ Bruker MALDI Biotyper® System. The *E. coli *susceptibility profile was obtained using the BD Phoenix™ automated identification and susceptibility testing system. On day three, vancomycin was discontinued, and meropenem was de-escalated to ceftriaxone when the susceptibility profile was available. The urine culture collected during admission was negative; however, the previous hospitalization urine culture showed an *E. coli* isolate with a very similar susceptibility profile to the blood isolate (Table 2).

**Table 2:** Blood and urine cultures susceptibility profile and interpretation S: susceptible, I: intermediate, R: resistant, MIC: minimum inhibitory concentration

	Current Hospitalization Blood Culture		Previous Hospitalization Urine Culture	
	Interpretation	MIC (µg/mL)	Interpretation	MIC (µg/mL)
Amikacin	S	<=8	S	<=16
Amoxicillin/Clavulanic Acid	I	16/8	S	<8/4
Ampicillin	R	>16	R	>16
Ampicillin/Sulbactam			R	>16/8
Aztreonam	S	<=8	S	<=8
Cefazolin	I	16	S	<=8
Cefepime	S	<=8	S	<=8
Cefotaxime	S	<=2	S	<=8
Cefoxitin	S	<=8		
Ceftazidime	S	<=1	S	<=2
Ceftriaxone	S	<=8	S	<=8
Cefuroxime	S	<=4	S	<=4
Cephalothin			S	<=8
Ciprofloxacin	R	>2	R	>2
Ertapenem	S	<=2		
Gentamicin	S	<=4	S	<=4
Imipenem	S	<=1	S	<=1
Levofloxacin	R	>4	R	>4
Nitrofurantoin				<=32
Piperacillin	R	>64	R	>64
Piperacillin/Tazobactam	S	<=16	S	<=16
Tetracycline	S	<=4	S	<=4
Ticarcillin	S	<=16	S	<=16
Tobramycin	S	<=4	S	<=1
Trimethoprim				<=8
Trimethoprim/Sulfamethoxazole	S	<=2/38	S	<=2/38

Given active bacteremia, there were concerns for prosthetic valve infection, prompting further workup. A transthoracic echocardiogram (TTE) showed the bi-leaflet #27 St. Jude mechanical mitral prosthesis opening well with a normal gradient and trace regurgitation. New blood cultures were collected to ensure clearance of bacteremia on the current antibiotic regimen. On day four, transesophageal echocardiogram (TEE) 3D images showed large mobile echogenicities with frondlike attachments on the prosthetic valve along the anterior annulus. On day five, gentamicin was added to the antibiotic regimen after evaluation by the Infectious Diseases team. The cardiothoracic surgery team also evaluated the patient for possible valve surgery; however, they determined she was a high-risk surgical candidate and recommended medical management only. On day eight, new blood cultures collected were finalized as negative, and the patient was clinically better and hemodynamically stable. Therefore, she was transferred to a long-term acute care facility to complete a total of six weeks of intravenous gentamicin and ceftriaxone from the first day of negative blood cultures, and arrangements to follow up in the clinic were made. During her follow-up, she reported being symptom-free. A repeat TEE eight weeks after hospital discharge revealed the complete resolution of vegetations.

## Discussion

Prosthetic valve endocarditis (PVE) is a rare and life-threatening complication of valve replacement surgery associated with high morbidity and mortality, representing 10-30% of all cases of IE with an incidence of 0.3-1.2% per patient per year [7]. Although the PVE mortality rate has decreased from 60% to 30% over the last two decades, it remains high despite its early diagnosis, available antimicrobial therapy, and prompt surgical intervention [7].

In the International Collaboration on Endocarditis-Prospective Cohort Study registry, which included patients from 61 international collaborative hospitals, *E. coli *was the most common etiology among the non-HACEK GNR [2,3,8]. The registry included 2,761 patients with a definitive diagnosis of IE; 49 (1.8%) were caused by non-HACEK GNR [2,3,8]. *E. coli *accounted for only 14 (29%) cases in this group. There was a higher in-hospital mortality rate (24%) of non-HACEK GNR compared to that of their HACEK counterparts (4%) [3]. Thus, it is imperative to ensure prompt diagnosis and timely interventions to prevent complications. Over the last four decades, 34 cases of *E. coli* IE have been reported in the PubMed database [9,10], and only 10 of them (Table 3) were associated with a prosthetic valve (PV) [10-14]. Hence, this is the 11th report of this rare condition.

**Table 3:** E. coli PVE cases reported in the PubMed database since 2005

Author and Year	Age and Sex	Source	Prosthetic Valve Infected	Antibiotic Regimen and Duration	Surgery	Outcome
Branger [10] 2005	60 years female	Gastrointestinal	Aortic	Cefepime + ciprofloxacin six weeks	Yes	Alive
Branger [10] 2005	76 years male	Gastrointestinal	Mitral	Ciprofloxacin six weeks	No	Alive
Branger [10] 2005	66 years female	Genitourinary	Mitral	Imipenem + gentamicin two weeks followed by ceftriaxone four weeks	Yes	Alive
Branger [10] 2005	76 years female	Genitourinary	Mitral	N/A	N/A	N/A
Modi [11] 2011	62 years female	Unknown	Mitral	Imipenem six weeks	No	Alive
Senel [12] 2012	60 years male	Gastrointestinal	Aortic	Ampicillin/sulbactam + gentamicin six weeks	Yes	Alive
Loubet [13] 2015	82 years N/A	Genitourinary	Aortic	N/A	Yes	Alive
Loubet [13] 2015	74 years N/A	Gastrointestinal	Mitral	Ceftriaxone + amikacin N/A	No	Alive
Loubet [13] 2015	74 years N/A	Genitourinary	Aortic	Ceftriaxone + ofloxacin six weeks	No	Death
Quiring [9] 2021	55 years Male	Gastrointestinal	Mitral	Ceftriaxone + gentamicin four weeks	No	Alive

The risk factors associated with *E. coli* IE include female sex, age greater than 70, previous healthcare exposure, implanted endovascular devices, diabetes mellitus, a genitourinary source of infection, and immunosuppressive therapy [2,3,6]. Of the 10 cases of *E. coli* PVE previously reported, the mitral valve was the most affected (60%) [10]. Our patient possessed several characteristics for developing *E. coli* IE, including a mechanical mitral valve, age, female sex, uncontrolled diabetes mellitus, previous hospitalizations, and recurrent UTIs. Imaging studies showed a non-obstructing kidney stone, multiple bilateral renal cysts, and endometrial thickening, which are abnormalities likely predisposing the patient to repetitive UTIs and consequent intermittent *E. coli* bacteremia, finally producing the seeding of the PV.

Before the TEE report of vegetations in the PV, our patient had already fulfilled the diagnosis of possible IE using the modified Duke’s criteria [2]. She met three minor criteria: (1) predisposing heart condition, i.e., prosthetic mitral valve, (2) fever, and (3) microbiological evidence, i.e., positive blood cultures with an organism not meeting the major criterion. The positive TEE for IE constituted the major criterion to complete the clinical criteria requirements: three minor criteria and one major criterion for a definite diagnosis of IE [2].

TEE should be performed first when evaluating patients suspected of PVE as it is more sensitive than TTE detecting vegetations and abscesses [2]. In the presence of a PV, TTE will not definitely rule out IE or its potential complications [2]. TEE better sensitivity was evident in our patient by an initial negative TTE and a positive TEE for vegetations. This difference is because TTE images are greatly hampered by the structural components of the prosthesis and are inadequate for assessing the perivalvular area [2]. Although a TEE should be performed first in patients suspected of PVE, limiting factors prevent its application, such as patients not fasting for at least six hours before the procedure and the absence of 24-hour TEE services [2]. Our patient had the TTE performed first as it is readily available in our institution, and a TEE needs up to 24 hours for coordination.

With the lack of robust data to define the optimal antimicrobial regimen to treat PVE caused by non-HACEK GNR, managing such cases can be challenging. The American Heart Association (AHA) considers it reasonable to use combination antibiotic therapy to manage native valve endocarditis with a β-lactam plus either an aminoglycoside or a fluoroquinolone for six weeks [2]. However, no recommendations are provided to treat PVE by non-HACEK GNR. Multiple antibiotic resistance mechanisms in non-HACEK GNR, such as inducible β-lactamases, become a limiting factor in managing IE, warranting infectious diseases consultation [2]. Morpeth et al. reported a similar mortality rate of 25% in patients with non-HACEK GNR who did and did not receive surgical intervention [3]. This observation suggests that IE caused by non-HACEK GNR is not an absolute indication for surgery. Consistent with the suggested management by the AHA, our patient received combination antibiotic therapy, leading to the resolution of vegetations from the PV and clinical improvement of symptoms.

## Conclusions

Although *E. coli* PVE is a relatively rare disease, it is associated with a high in-hospital mortality rate and, thus, requires prompt diagnosis and intervention. In patients with persistent or intermittent *E. coli* bacteremia who possess predisposing factors, physicians should have a high index of suspicion for IE and consider obtaining appropriate diagnostic testing. Management can be challenging due to the lack of specific treatment recommendations. Hence, there is a need for prospective studies to ascertain the best treatment.
